# Ectopic expression of human acidic fibroblast growth factor 1 in the medicinal plant, *Salvia miltiorrhiza*, accelerates the healing of burn wounds

**DOI:** 10.1186/1472-6750-14-74

**Published:** 2014-08-09

**Authors:** YaQing Tan, Kevin Yueju Wang, Nan Wang, GangQiang Li, DeHu Liu

**Affiliations:** 1 Biotechnology Research Institute, Chinese Academy of Agricultural Sciences, 12 Southern Zhong Guan Cun Road, Beijing 100081, China; 2 Department of Natural Sciences, Northeastern State University, Broken Arrow, Oklahoma 74014, USA

**Keywords:** Human acidic fibroblast growth factor 1, Medicinal plant, *Salvia miltiorrhiza*, Burn wound healing

## Abstract

**Background:**

Healing of burns is a complex process and very few effective treatments exist to facilitate the burn recovery process. Human acidic fibroblast growth factor 1 (FGF-1) plays an important role in a variety of biological processes, including angiogenesis, and tissue repair. *Salvia miltiorrhiza* is widely used in traditional Chinese medicine as an herb for the treatment of various diseases, including cardiovascular and cerebrovascular diseases, and traumatic injuries. We present that expression of FGF-1 in *S. miltiorrhiza* significantly accelerates the healing of burn wounds.

**Results:**

The human *fgf-1* gene was fused with a barley α-amylase signal peptide DNA sequence and driven by a 35S promoter for constitutive expression in transgenic *S. miltiorrhiza* plants*.* The highest yield of recombinant FGF-1 obtained from leaves of transgenic *S. miltiorrhiza* lines was 272 ng/fresh weight*.* Aqueous extracts from transgenic *S. miltiorrhiza* exhibited FGF-1 activity approximately 19.2-fold greater than that of the standard FGF-1. Compared to the standard FGF-1 or the extracts obtained from non-transgenic plants, it stimulated proliferation of Balb/c 3 T3 mouse fibroblast cells assessed with the standard MTT assay and promoted angiogenesis in the chicken embryo chorioallantoic membrane (CAM) assay. Topical application of the extract significantly accelerated the burn wound healing process.

**Conclusions:**

The product appears to retain the biological activity of both FGF-1 as well as the medicinal properties of the plant. The extracts from transgenic *S. miltiorrhiza* combines the therapeutic functions of FGF-1 and the medicinal plant, *S. miltiorrhiza*. Topical application of the product can reduce the costs associated with extraction, purification, and recovery.

## Background

Various factors, such as heat, electricity, UV-light, and chemicals, can cause burns. Although most burns are not fatal, over 300,000 deaths occur globally each year as a direct result of burns, with 90% of burn deaths occurring in developing countries [1]. Healing of burns is a complex process and very few effective treatments exist to facilitate the burn recovery process. Antibiotics are often used to prevent or treat infections and skin or synthetic grafts are used to facilitate the healing process in cases of third-degree burns [2].

Fibroblast growth factors (FGFs) comprise a large family genes involved in growth and differentiation. They are found in both invertebrates and vertebrates [3,4]. FGFs have been demonstrated to play essential functions in development, metabolism, and repair of various tissues and organs. Human FGFs contain 22 family members. Human FGF-1 is one of the most studied and characterized members of the superfamily. FGF-1 has various biological functions and has been associated with various stages of morphogenesis, tissue repair, angiogenesis and wound healing [3,4].

FGF-1 is present in various tissues, especially within the extracellular matrix, however, isolating and purifying this protein is a complicated and tedious process. Recombinant DNA technology has enabled FGF-1 to be produced in *E.coli*[5], yeast [6], mammalian cells [7] and plants [8] for potential medical applications. Most studies designed to evaluate its effect on wound healing and tissue regeneration have administered recombinant FGF-1 directly to animal [9,10] or human wound sites [10]. Like other FGFs, however, FGF-1 has low stability and is very sensitive to degradation by proteases [3,4]. Unfortunately, recombinant FGF-1 exhibits a very short half-life *in vivo* which limits its medical application.

*Salvia miltiorrhiza*, also known as Danshen, native to China and Japan, is a traditional Chinese medicinal (TCM) herb. The extract of its root has been widely used clinically to treat and prevent diseases, such as cardiovascular disease, hyperlipidemia, cancer, liver fibrosis and cirrhosis, etc. [11]. Leaf extract of *S. miltiorrhiza* also contains considerable amounts of pharmacological compounds even it is discarded as waste during root harvest. More than seventy compounds from *S. miltiorrhiza* have been investigated. Three major bioactive compounds, tanshinone-I, tanshinone-IIA, and cryptotanshinone have been extensively studied and reported to have a variety pharmacological activities, including anti-inflammatory [12], antioxidant [13], antitumor [14] and anti-platelet aggregation [15]. TCM compendiums indicate that *S. miltiorrhiza* reduces blood stasis by promoting blood circulation and repairing damaged tissues. The use of *S. miltiorrhiza* to treat coronary heart disease and hypertension, as well as for stroke patients, is in clinical phase IV trials (clinicaltrials.gov). However, like many TCM herbs, the precise mechanism underlying the biological activity of *S. miltiorrhiza* still remains to be completely elucidated.

In the present study, FGF-1 was constitutively expressed in the medicinal plant, *S. miltiorrhiza*. Presence and levels of FGF-1 were analyzed by ELISA and Western blot. Aqueous extracts of transgenic *S. miltiorrhiza* were applied directly to fertilized chicken egg embryo chorioallantoic membranes (CAM) and to second degree burn wounds on Sprague Dawley (SD) rats. Results indicated that the transgenic plants exhibited a both the biological function of FGF-1 and the medicinal properties of *S. miltiorrhiza*. Data indicated that the extract obtained from the transgenic plants could promote cell proliferation, speed up the growth of new blood vessels and significantly accelerate the burn wound healing process compared to the use of either FGF-1 or *S. miltiorrhiza* alone. The use of the transgenic plant extract also avoids the complicated process of FGF-1 extraction and purification.

## Results

### Gene cloning and plant transformation

**Figure 1:**
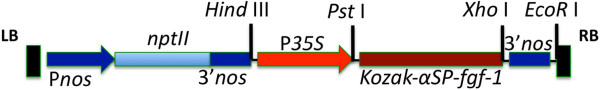
**Plant expression construct pBI121- *α *FGF.** The construct was made in the T-DNA vector, pBI121. The coding region of *fgf-1* was fused with an alpha amylase signal (*αSP*). A Kozak sequence was inserted before the start codon. *Pnos*, promoter from nopaline synthase gene; nptII, neomycin phosphotransferase II gene; *3’nos*, nopaline synthase terminator; *p35S*, *CaMV 35S* promoter. LB and RB, left and right border T-DNA sequence. *Hind* III, *Pst* I, *Xho* I and *EcoR* I were restriction enzymes used for cloning FGF-1.

Human FGF-1 was cloned into the expression vector, pBI121, with a Kozak consensus sequence inserted before the start codon and a barley alpha amylase signal sequence at the 5′ end, driven by a cauliflower mosaic virus 35S (CaMV35S) promoter (Figure 1) for strong constitutive expression. At least 120 independent putative transgenic events were obtained. All putative transformants were analyzed by PCR amplification using P1 and P2 primers. Results indicated that fifty-six of the putative transformants had the predicted 0.5 kb band.

### ELISA and western blot analysis

The level of rFGF-1 produced in the confirmed transgenic lines was determined by ELISA. Results revealed that the yield of rFGF-1 in lines T65, T97 and T117 were131, 146 and 272 ng/g fresh leaf weight, respectively, as calculated by comparison with a standard curve constructed using commercial FGF-1 (see Additional file 1: Table S1).

**Figure 2:**
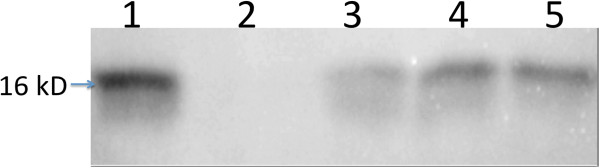
**Western immunoblotting assay.** Standard FGF-1 (Lane 1), rFGF-1 expression in wild type plants (Lane 2), and transgenic plant lines, T65 (Lane 3), T97 (Lane 4) and T117 (Lane 5). 10 μL of supernatant from each sample was used in the immunoblotting assay.

Recombinant human acidic fibroblast growth factor 1 (rFGF-1) protein expression in transgenic lines T65, T97 and T117 was determined by the Western blot analysis (Figure 2). A positive response using FGF-1 monoclonal antibody demonstrated that transgenic lines (T65, T97, and T117) had a 16 kDa band corresponding in size to standard FGF-1. No band was observed in protein samples extracted from WT plants. The results of the Western blot also indicated that T117 had a higher level of expression than T65. It corresponded with the results obtained in the ELISA assay. Since T117 had the highest level of rFGF-1, it was examined further. Transgene copy number in line T117 was determined by Southern blot analyses (see Additional file 2: Figure S1). Results indicated that there was a single T-DNA copy integrated in line T117.

### Proliferative effects of transgenic *S. miltiorrhiza* (T-SM) on cell proliferation *in vitro*

**Figure 3:**
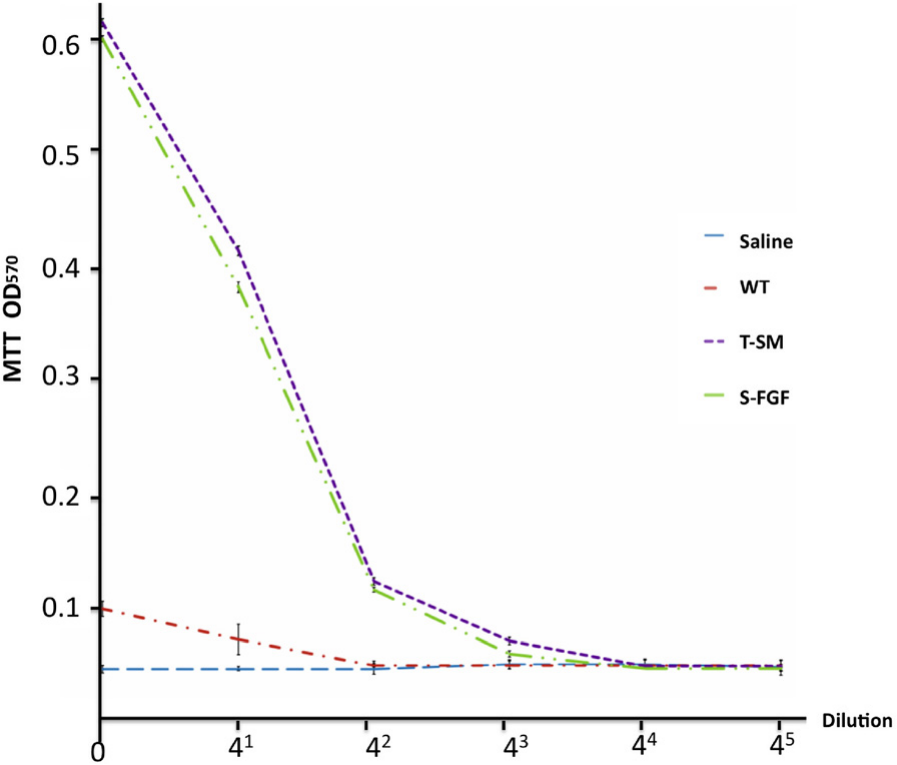
**MTT assay for Balb/c 3 T3 cell proliferation analysis.** After 72 h treatment, the proliferation rate in the extract of transgenic *S. miltiorrhiza* plant (T-SM) was significantly higher compared to the standard FGF-1 (S-FGF), wild-type plant (WT) and saline solution ( *p* ≤ 0.001). Data for 4 replicates and 3 repeats were averaged, and error bars represent SD.

After 72 h of culture, MTT assay revealed that wild-type *S. miltiorrhiza***(**WT), standard FGF-1 (S-FGF) and T-SM significantly promoted Balb/c 3 T3 cell proliferation compared to the Saline control (Figure 3). The viability of the cells decreased when the cells were treated with diluted concentrations of samples. After the second (4^2^ fold) dilution, WT showed had no effect on cellular viability. After the third (4^3^ fold) dilution, the absorbance of T-SM (0.076 ± 0.0034) was still significantly higher than that of S-FGF (0.065 ± 0.0026). The results indicated that treatment with T-SM greatly increased the cell proliferation compared with that of the other groups. The estimated effective dose (ED50) stimulating 50% of the maximal cell number response was determined from MTT proliferation curves. The dilution fold of ED50 for S-FGF and T-SM were 4^1.5^ and 4^1.47^, respectively. The converted enzyme activity of T-SM (4.8×10^6^ IU/mg) was approximately 19.2-fold greater than that of the standard FGF-1 (2.5 × 10^5^ IU/mg) (Table 1).

**Table 1:** **Enzymatic activity of the recombinant FGF-1 from transgenic *S. miltiorrhiza***

	**FGF-1 content**	**ED50 (dilution)**	**AU/mL**	**U/mg**
S-FGF	200 ng/mL	4^1.5^	50	2.5 × 10^5^
T-SM	10 ng/mL	4^1.47^	48	4.8 × 10^6^

Effective dose 50 (ED50) is determined by MTT assay curve using Balb/c 3 T3 cells. The dilution fold is used to determine that compounds are active at that concentration to proliferate 50% of the cell growth. The enzymatic activity of recombinant FGF-1 from transgenic *S. miltiorrhiza* (T-SM) is determined in terms of AU/mL in relation to a standard human acid FGF-1 (S-FGF) with a known activity (50 AU/mL). The specific enzymatic activity Units of FGF in T-SM was calculated as 48 AU/mL (4^1.47^/4^1.50^ × 50 AU/mL), with a conversion of (1000 × 1000/10 ng) × 48 = 4.8 × 10^6^ U/mg.

### T-SM stimulates angiogenesis *in vivo*

**Figure 4:**
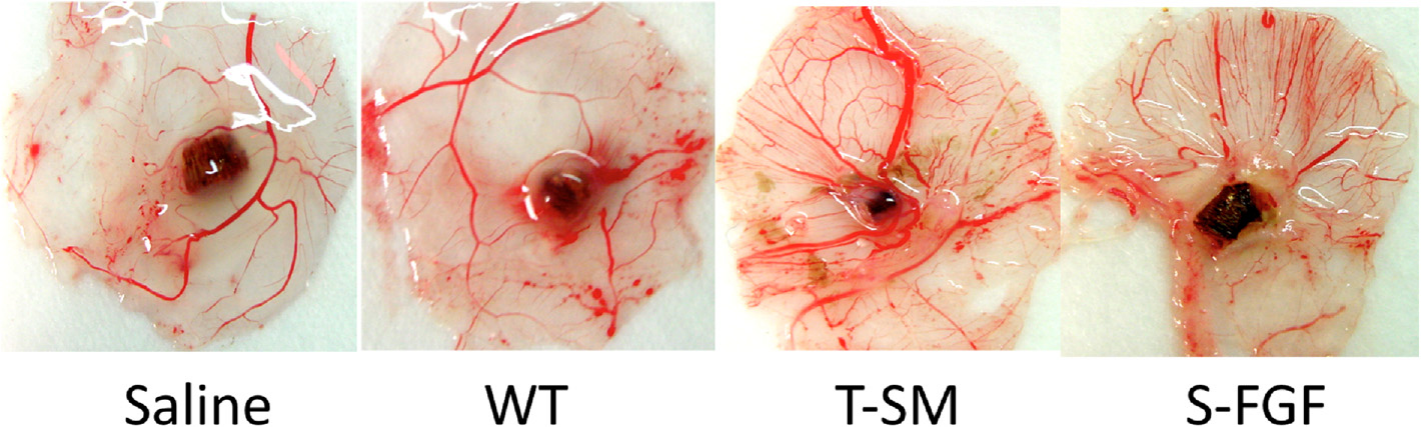
**Extracts from transgenic plant promotes angiogenesis in the CAM assay.** A: Saline solution was used as negative control. 5 μL of aqueous extract from wild-type *S. miltiorrhiza* plants (WT) and transgenic *S. miltiorrhiza* (T-SM). S-FGF treatment contained 50 ng of FGF-1 produced in *E. coli* and obtained commercially.

The CAM assay is widely used to study angiogenesis *in vivo*. Our study demonstrated that treatment with extracts from WT, S-FGF and T-SM all stimulated angiogenesis (Figure 4). At day 11, a very few large vessels with only few branching were observed with in the samples treated with just saline. In comparison to the saline treatment, a greater number of large vessels and branches were formed in the WT treatment. T-SM treatment induced a significantly greater number of large vessels and branches compared to all of the other treatments. Even though the concentration of T-SM rFGF-1 used in the CAM assay was 1/18.5 of the S-FGF treatment (≈2.7 ng vs. 50 ng, respectively), it stimulated more vessels. The greatest amount of branch formation was also observed with T-SM-induced angiogenesis in the CAM assay.

### T-SM Accelerates wound healing process

**Figure 5:**
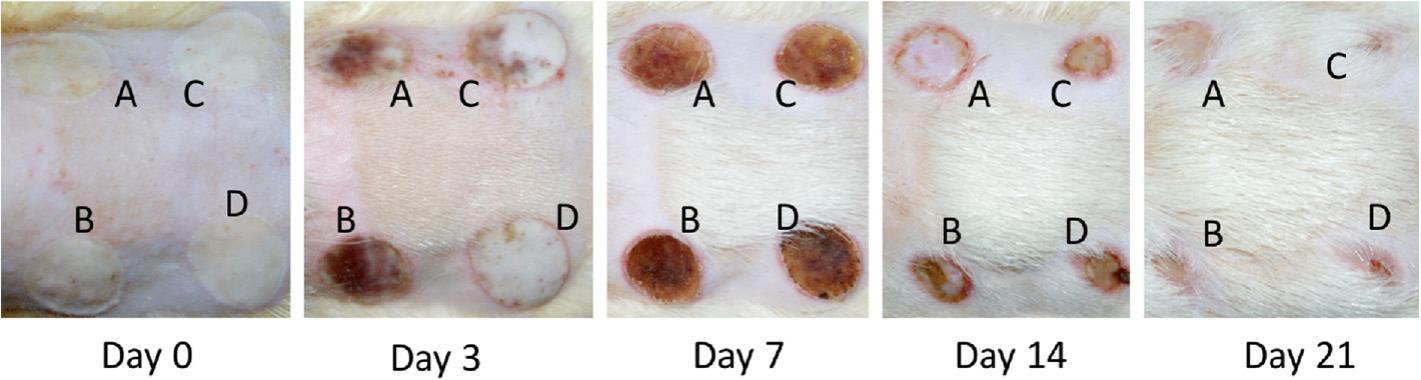
**Extracts from transgenic plants accelerate the burn wound healing process.** Second degree burn wounds were administered on the dorsal skin of SD rats. The area of the burn wounds was treated with **(A)**, 100 μL of saline **(B)** aqueous extract from wild type plants; **(C)**, 1000 ng of commercial FGF-1, and; **(D)** 100 μL of aqueous extract from transgenic *S. miltiorrhiza*. Photographs were taken on Day 0, 3, 7, 14 and 21 after burn injury was administered.

**Figure 6:**
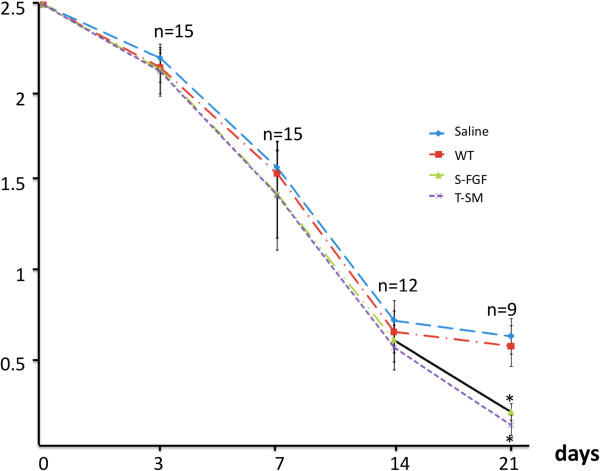
**Burn wound surface assay.** Area as evaluated using the transparent tracing method described by Bohannon and Pfaller (1983). Wounds were treated with either 100 μL of saline, 1000 ng commercial FGF-1, or 100 μL aqueous extract from wild type or transgenic *S. miltiorrhiza*. Burn wound area was recorded on day 3 (n = 15), 7 (n = 15), 14 (n = 12) and 21 (n = 9). The data represent the Means ± se. n: rats number for each treatment. *indicates significant difference (P < 0.01) between the specified treatment and the saline treatment.

Images of burn wounds treated with saline, WT, S-FGF (FGF-1 1000 ng/wound), and T-SM (rFGF-1 ≈ 2.7 ng/wound) at 0, 3, 7, 14, and 21 d after administering the burn treatment are shown in Figure 5. On day 3, shrinkage of the wounds was observed in all groups. WT and saline groups had begun to form dark granulation tissue. In contrast, T-FGF-1 treated wounds appeared smooth and whitish. FGF-1 treated wounds were also smooth but exhibited some granulation tissue. On day 7, wounds in all groups continued to shrink and develop thick granulation tissue. On day 14, while considerable contraction of wounds was observed in all treatments, T-SM treated wounds began rebuilding healthy granulation tissue. On day 21, the S-FGF and T-SM treated wounds were almost healed, while WT and saline treated wounds were still very visible and whitish in appearance. Wound surface area tracing measurements (Figure 6) did not identify significant difference among the four treatments on day 0, 7 and 14 and also revealed that the healing process appeared normal. On day 21, however, both T-SM and S-FGF treated wounds were significantly reduced (P < 0.01) in wound surface area (0.14 ± 0.06 and 0.21 ± 0.05, respectively) compared to the saline (0.64 ± 0.10) and WT (0.58 ± 0.11) treated wounds. S-FGF and T-SM treated wounds were almost completely healed. No difference in wound surface area was observed between saline and WT treated burns. Importantly, while the concentration of FGF-1 in the S-FGF treatment was 1000 ng FGF-1/wound, it was only ≈ 2.7 ng rFGF-1 in the T-SM treatment. However, they exhibited a similar ability to accelerate the wound healing process.

### Histopathological assessments of burn wound healing process

**Figure 7:**
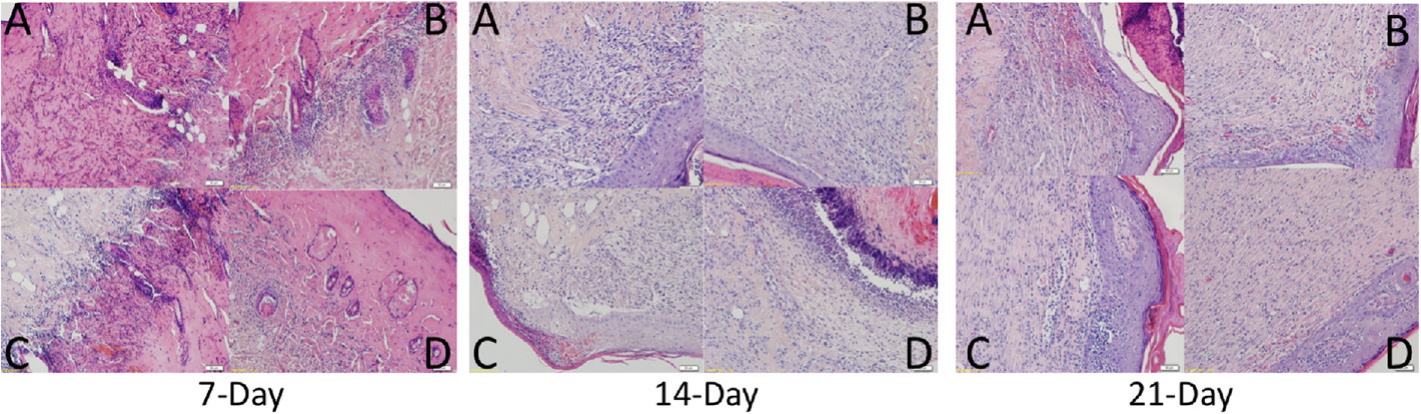
**H&E staining.** Cryosections (2 μm thick) stained with hematoxylin (H) and eosin (E) to examine the burn wound healing process after application of different treatments. Samples were taken on day 7, 14 and 21. **A**: 100 μL of saline; **B**: 100 μL saline aqueous extract from wild type (WT) *S. miltiorrhiza;***C**: 1000 ng FGF-1; and **D**: 100 μL saline aqueous extract from transgenic *S. miltiorrhiza*. Micrographs were taken at 200×.

Histopathological analysis of normal tissues and the burn wound healing process was conducted by sectioning of tissues and staining with hematoxylin and eosin staining (H & E). A standard second degree burn wound was established (see Additional file 3: Figure S2). Normal skin had an intact cuticle, hair follicles, sebaceous glands, collagen and a prickle cell layer (A and B). Epidermal necrolysis was observed 24 h after burn treatment (C and D). In the dermis, dead cells disintegrated and collagen fibers became disorganized. Hair follicles were also damaged and vasodilation and congestion of the blood vessels were evident.On day 7, the saline treated burn wounds exhibited inflammatory cells in epidermal necrolysis tissue. An inflammatory response in the wound area was not obvious (Figure 7). In other treatments, homogeneous necrosis was evident in the epidermal exudate of burn wounds but with less inflammatory cells. The inflammatory response in the wound area was obvious with an increased number of inflammatory cells (B, C, D). On day 14, in contrast to saline treated wounds, the burn wounds in all other treatments exhibited rapidly growing granulation tissue, including fibroblasts, capillaries, and inflammatory cells. In S-FGF and T-SM treated burn wounds, a greater amount of newly formed epidermis began to migrate into the wound area (C and D). On day 21, saline treated burn wounds still exhibited a thick surface layer of dead cells. A large number of inflammatory cells were also observed with some bleeding near the epidermis. An incomplete monolayer of epidermal cells was also observed. Burn wounds in all the other treatments exhibited good re-epithelialization. A thicker prickle cell layer was observed in S-FGF treated wounds than in T-SM treated wounds. Tissue directly in the wound area began to form hair follicles and other skin appendages. T-SM treated wounds exhibited less necrotic tissue compared to all the other treatments.

## Discussion

Our studies demonstrated that the medicinal plant, *S. miltiorrhiza*, can be used to produce functional human FGF-1. In addition to acquiring the therapeutic function of FGF-1, the transgenic plants also contained natural, endogeneous, medicinal compounds, such as tanshinone-I, tanshinone-IIA and cryptotanshinone. The therapeutic effect of the transgenic plants was significant and greater than using either FGF-1 or the medicinal plant alone. Western blot (Figure 2) assays indicated that FGF-1 was expressed in the transgenic *S. miltiorrhiza*.

The highest accumulation of recombinant FGF-1 level in transgenic *S. miltiorrhiza* was 272 ng/g fresh leave weight in line T117. We found that topical application of crude extracts from transgenic *S. miltiorrhiza* could enhance angiogenesis and accelerate the burn wound healing process.

The drawback in using plants to produce recombinant proteins is that plant cells often recognize the proteins as foreign and target them for degradation. Since only the expected single band was observed in the western blot assay, this indicated that the rFGF-1 in *S. miltiorrhiza* had not been subjected to proteolysis. Reports have suggested that foreign gene expression, and protein yield in transgenic plants could increase after producing several generations (T1-T4) of plants from self-pollinated primary (T0) transgenic lines [16,17]. We selected the line T117, which has a single transgene integration event as determined by Southern blot (Additional file 2: Figure S1), to produce subsequent generations in order to determine if the yield of rFG-1 in transgenic *S. miltiorrhiza* can be increased.

Extracts of non-transgenic *S. miltiorrhiza* can inhibit the stickiness of blood platelets and decrease fibrin [11,15]. The extract from this plant is widely used in traditional Chinese medicine to treat cardiovascular and cerebrovascular diseases [11]. One of its compounds, salvianolic acid, was reported to enhance *in vitro* angiogenesis in rat endothelial cells through the up-regulation of VEGF and VEGF receptors genes that promote cell growth and differentiation [18]. In the CAM assays, we also observed that the *S. miltiorrhiza* treatment produced more blood vessels (Figure 4). These results indicate that *S. miltiorrhiza* may facilitate angiogenesis. Very few studies have reported that *S. miltiorrhiza* has an effect on the burn wound healing process. One study indicated that injection of *S. miltiorrhiza* and ligustrazine can effectively reduce myocardial damage in patients with severe burns [19]. Animal studies also indicate that *S. miltiorrhiza* enhances the wound healing process [20,21]. Randomized clinical trial of *S. miltiorrhiza* suggested that it is effective in reducing skin flap ischaemia necrosis after mastectomy [20]. *S. miltiorrhiza* can stimulate the cell proliferation and it has the potential to be used for wounding healing or cosmetic treatment [22]. In this study, the *S. miltiorrhiza* treatment stimulated fibroblast cell proliferation (Figure 3). The burn wound healing process is complex. We did not observe that *S. miltiorrhiza* treatment of burn wounds reduced burn wound area compared to the saline treatment (Figures 5 and 6). H&E staining, however, indicated that *S. miltiorrhiza* treatment can promote the formation of new cells and tissues to accelerate wound re-epithelialization. *S. miltiorrhiza* can promote the circulation system in wounded tissue (Figure 7). It may improve the burn wound healing process by removing necrotic cells, attenuating the inflammatory response and apoptosis [12,21], reducing oxidative stress [12], and possibly facilitate the repair of tubular epithelia structures [23].

The function of FGFs in tissue repair, angiogenesis and wound healing is well documented [3,4]. FGF-1 and FGF-2 promote cell proliferation and migration, as well as induce the physical organization of endothelia cells into blood vessel structures [24,25]. These properties can facilitate the burn wound healing process. The MTT, CAM assays, and burn wound healing test used in the present study also demonstrated that the application of FGF-1 enhanced cell proliferation and induced a greater number of new blood vessels and reduced the burn wound surface area more than treatments in which FGF-1 was absent (Figures 3, 4 and 5). These results were further supported by the histological analysis. The healing rate in the S-FGF treated wounds was more rapid compared to either the saline or WT treated wounds. The healing rate in S-FGF and T-SM treated burn wounds was similar. H&E staining revealed less necrosis in T-SM treated wounds than in S-FGF treated wounds. Approximately 2.7 ng of the rFGF-1 was used in the T-SM treatment while 1000 ng FGF-1 was used in the S-FGF treatment. This indicates that the T-SM treatment was significantly more efficient in its effect on angiogenesis and the burn wound healing process. The FGF-1 used in the S-FGF treatment was produced in *E.coli*. Common microbial hosts such as *E. coli* can produce high yields of recombinant protein but lack the requisite machinery for post-translational modification required for protein stability and bioactivity [26]. In contrast, the protein synthesis process in plants is very similar to animal cells. The structure and function of rFGF-1 derived from transgenic *S. miltiorrhiza* may be more similar to native, human FGF-1 and thus have higher activity than FGF-1 obtained from *E.coli. S. miltiorrhiza* exhibits anti-inflammatory [12], antioxidant [13] and anti-bacterial activity [27]. The medicinal properties of *S. miltiorrhiza* combined with FGF-1 functions appears to accelerate burn wound healing by improving blood circulation and providing more nutrients and oxygen to the wound area.

FGFs are readily degradable *in vivo*, which results in a loss of biological activity and function [3,4,28,29]. FGF-1 biological activity was maintained in the extracts obtained from transgenic *S. miltiorrhiza* and applied topically to burn wounds. *S. miltiorrhiza* is a medicinal plant and contains natural compounds that also have various pharmaceutical functions. Overexpression of *fgf-1* in *S. miltiorrhiza* provides the benefits of both FGF-1 and the medicinal properties of the plant, which may enhance its therapeutic effects. Plant specific glycans can harm humans and injection of recombinant plant therapeutic proteins may result in an immunogenic response in humans [30]. Topical application of transgenic *S. miltiorrhiza* extracts avoids plant-specific glycosylated immunogenic concerns and reduces the complex and costly purification and recovery process associated with the production of therapeutic compounds in microorganisms.

## Conclusions

We successfully overexpressed *fgf-1* in transgenic *S. miltiorrhiza*. Supernatant from homogenated transgenic *S. miltiorrhiza* plants promoted angiogenesis and accelerated the burn wound healing process. The product system combines the therapeutic functions of FGF-1 and the medicinal plant, *S. miltiorrhiza*. Topical application of the product can reduce the costs associated with extraction, purification, and recovery. The yield of rFGF-1 could be further increased by optimizing the gene codon, targeting the protein to the specific subcellular compartments, or selfing of high-expressing lines to obtain homozygous plants. The mechanism associated with the enhanced biological activity of *S. miltiorrhiza* and FGF-1 combined in the same extract will require further study.

## Methods

### Materials

The plasmids, pUC-haFGF (containing *fgf-1* gene), pTΩ4A, the plant expression construct pBI121, and *Agrobacterium tumefaciens* LBA4404 are materials maintained in our lab. Seeds of *S. miltiorrhiza* were purchased from Shangluo Northwestern Medical Plant Company of Shaanxi (Shaanxi, China). Balb/c 3 T3 mouse fibroblast cells were bought from the Cell Culture Center, Institute of Basic Medical Sciences of Chinese Academy of Medical Sciences and School of Basic Medicine of Peking Union Medical College (Beijing, China). Fertilized eggs were obtained from the Institute of Animal Sciences (IAS), Chinese Academy of Agricultural Science (CAAS), Beijing, China. Male Sprague–Dawley (SD) rats (aged 6 to 7 weeks) were purchased from Vital River Laboratories Company (Beijing, China). All animal protocols were approved by the CAAS Institutional Ethics Committee.

### Plasmid construction

In order to enhance recombinant FGF-1 expression level, a Kozak consensus sequence was inserted before the start codon and a barley alpha amylase signal peptide [31,32] was fused to the FGF-1 sequence for extracellular targeting of the recombinant FGF-1. Three rounds of PCR were performed. The first round of PCR used P1 and P2 primers. P1 (forward): 5′-*CTTTCTGCCAGCTTGGCCTCCGGACAAGTT*TACAAGAAGCCAAAGTTGCTTTACT-3′ (Italicized sequences indicate the partial 3′- end alpha amylase signal peptide sequence obtained from barely (*Hordeum vulgare*) and P2 (FGF-1 reverse); 5′-AA*CTCGAG*TTAATCAGAAGAAACTGGCAAT-3′ (*Xho* I site is italicized)*.* PCR product resulting from the use of the P1 and P2 primers was used as the template for the second round PCR with P3 and P2 primers. P3 (forward):5′-*TCCCTCTCCCTCTTCCTCGTCCTCCTTGGC*CTTTCTGCCAGCTTGGCCTCCGGAC-3′ (Italicized sequences indicate the middle region of the alpha amylase signal peptide sequence). The second round PCR product resulting from the use of the P3 and P2 primers was used as the template for the third round PCR with P4 and P2 primers. P4 (forward): 5′- AA*CTGCAG***AACA**ATG*GCGAACAAACATTTG*TCCCTCTCCCTCTTCCTCGTCCTCC (Italicized sequences indicate the *Pst* I site and 5′-end partial alpha amylase signal peptide sequence, respectively. The bold indicates the Kozak sequence). The third round PCR product was analyzed on a 1% agarose gel and subcloned into a TA cloning vector. After sequence verification (TaKaRa, Dalian, China), the product was digested using *Pst* I/*Xho* I and inserted between the *35S* promoter and *Nos* terminator of pTΩ4A. The resulting *35S*-(*fgf-1*)-*Nos* cassette was digested using *Hind* III/*EcoR* I and then inserted into a plant binary vector, pBI121 using the same sites, to generate the plant expression construct, pBI121-aFGF.

### Plant transformation and selection

Transformation of *S. miltiorrhiza* utilized the method described by Horschet et al. [33] with modification. The binary vector pBI121-aFGF described above was introduced into *A.tumefaciens* strain LBA4404 using the triparental mating method. The resulting culture was plated on YEP (Yeast extract 10 g/L, Peptone 10 g/L, NaCl 5 g/L, pH 7.0) plates containing selective antibiotics (kanamycin 50 mg/L and rifampicin 50 mg/L). A single colony was analyzed by PCR using P1 and P2 primers, inoculated into 50 mL of YEB medium containing kanamycin (50 mg/L) and rifampicin 50 mg/L, and grown at 28°C, 240 rpm until the OD_600_ reached 1.0. The culture was pelleted by centrifugation and re-suspended in MS liquid medium to obtain an approximate OD_600_ of 0.5. Explants (0.5 cm × 0.5 cm) were excised from 2- to 3-week-old sterile *S. miltiorrhiza* seedlings and immersed for 5 to 8 min in the *Agrobacterium* suspension described above for 5 to 8 min. The explants were then blotted on sterile filter paper and plated on a co-cultivation medium (MS, 6-BA 2.0 mg/L) in the dark for 2 days at 25°C. After co-culture, the explants were transferred onto selection medium (MS, 6-BA 1.0 mg/L, kanamycin 50 mg/L, cefotaxime 400 mg/L). Cultures were incubated at 25°C/23°C (day/night temperature) with a 16-hr photoperiod. Explants were transferred to fresh selection medium every 2 weeks to generate shoots. Shoots were then transferred to a rooting medium (1/2 MS, sucrose 2.0%, kanamycin 30 mg/L) to obtain roots. Plants were acclimated for two weeks in vermiculite with half strength MS liquid medium and then transferred to soil.

### Southern blot analysis of putative transgenic plants

Putative transgenic plants were analyzed by DNA hybridization. Total leaf genomic DNA was extracted according to Minas et al. [34]. Thirty μg DNA was digested overnight with single site *EcoR* I or *Hind* III restriction enzymes. The digested product was run on a 0.8%agarose gel. Gel denaturation and neutralization was followed by a routine DNA Southern Blot [35]. The gel was blotted on positively charged nylon membranes by capillary transfer with 20 × SSC following the protocol described in the instruction manual for the DIG High Prime DNA labeling and detection kit (Roche Diagnostics, Indianapolis, USA). DNA fixation, hybridization and wash procedures also followed the manufacturer’s instructions. DIG-labeled probe was amplified with *fgf-1* specific primers P1 and P4. Immunological detection was performed according to the manufacturer’s instructions.

### ELISA and immunoblot analysis

The ELISA procedure was performed according to the method described in the instruction manual of the Human FGF-acidic ELISA Construction Kit (Antigenix America, Huntington Station, USA). Standard FGF-1 produced in *E.coli* (Wanxing-Bio, Shanghai, China) was used to create a series of dilutions (0, 3.125, 6.25, 12.5, 25, 50 and 100 ng/mL) in 1XPBS (phosphate buffered saline) buffer to construct a standard curve. Total soluble protein extracts were made by grinding 20 mg of fresh leaf material in 200 μL1XPBS buffer. The mixture was centrifuged at 12, 000 g for 10 min at 4°C. Recombinant FGF-1 (rFGF-1) protein present in 100 μL supernatant was loaded in duplicate wells of a microtiter plate and the OD_650_ was obtained. The concentration of rFGF-1 was determined by comparison with the constructed FGF-1 standard curve.

For immunoblot analysis, 50 mg of leaf disks were ground in 200 μL of deionizer water. The extract was centrifuged at 12, 000 g for 10 min at 4°C. 100 μL of supernatant was collected and concentrated to 20 μL at 4°C for 5 min using a vacuum concentrator (SPD1010 SpeedVac Syetem, Thermo Scientific, USA). 10 μL of the concentrated supernatant was separated by 15% SDS-PAGE, and transferred to a nitrocellulose membrane with an iBlot^®^ blotting system (Invitrogen). Western blot analysis of rFGF-1 was performed as described by Li et al. (2012). FGF-1 antibody (Sino Biological Inc. Beijing, China) was used as the primary antibody. Nonspecific binding was blocked by incubation in 3% bovine serum albumin (BSA) in TBST (25 mM Tris–HCl, pH 7.4, 0.14 mM NaCl, and 0.05% Tween 20) for 2 h at 4°C. The membrane was washed three times for 10 min each in TBST and then incubated in streptavidin-HRP for 1 h. The membrane was then wash three times in TBST and then stained with 4-Chloro-1-naphthol (4CN) to visualize the results.

### Cellular proliferation

The effect of wild-type *S. miltiorrhiza* (WT), transgenic *S. miltiorrhiza* (T-SM) or standard FGF-1 (S-FGF) on cell viability and proliferation was examined by thiazolyl blue tetrazolium bromide (MTT) assay described by Mosman [36]. Leaf tissue (0.25 g fresh weight) of WT and T-SM was ground in 0.75 mL of 1XPBS buffer respectively, and centrifuged at 12,000 g for 10 min at 4°C. The supernatants were diluted 5 times in RPMI 1640 medium supplemented with 0.4% fetal bovine serum (FBS). The yield of rFGF-1 in diluted aqueous extract from T-SM was 10 ng/mL determined by ELISA assay. S-FGF (200 ng/mL, enzyme activity unit: 50 AU/mL) was used as positive control. RPMI 1640 medium with 0.4% FBS was used as blank control. All samples (WT, S-FGF and S-FGF) were 4-fold serially (4°- to 4^5^- fold dilution) diluted in RPMI 1640 medium with 0.4% FBS.

A concentration of 6.5 × 10^3^ BALB/c 3 T3 cells per well were seeded in a 96-well microplate containing 100 μL of RPMI 1640 medium with 10% FBS. After 24 h of incubation in a humidified incubator with 5% CO_2_ at 37°C, all the medium was refreshed in 100 μL of RPMI 1640 with 0.4% FBS. The cultures were maintained for another 24 h. And then, the cells were exposed to 100 μL of sample solutions prepared previously with 100U/mL penicillin and 100 μg/mL streptomycin. After 72 h incubation, 25 μL of 5 mg/mL MTT (AMRESCO, USA) was added to each well. After 5 h treatment with MTT, 120 μL of 50% DMSO was added to the well and mixed thoroughly with pipette. The plate was shaken at room temperature 15 min. The absorbance of each well was determined at 570 nm using a microplate reader (Bio-rad, USA). Four replicates from each group were analyzed. The experiment was repeated 3 times. Results are expressed as mean ± standard error. Student’s *t* test was used to evaluate significance differences between groups at *p* < 0.05.

### CAM assay

The effect of various extracts on angiogenesis (blood vessel growth) was analyzed by a CAM assay using methycellulose disks described by Larger et al. [37]. Each disk was wetted with 5 μL of aqueous extract from either non-transgenic, wild-type *S. miltiorrhiza* (WT) or transgenic *S. miltiorrhiza* (T-SM), plants. 5 μL of a saline solution and 50 ng of standard FGF-1 (S-FGF) were used as negative and positive controls. 10 μL of 0.5% methylcellulose was dried in a well, 2 mm diameter and 0.6 mm deep, of culture plate. Forty five chick eggs were cleaned with a formalin solution and then incubated at 37°C and 75% RH. Abnormal embryo development was verified and the embryos with malformations or dead embryos were discarded. On day 9 of the incubation period, previously prepared saline, S-FGF, T-SM, or WT methylcellulose disks were placed on the CAM. The treated area was carefully excised after 48 h of incubation and washed with deionized water. The excised area was spread on 3MM filter paper and photographed with a Sony DSLR digital camera.

### Burn wound surface area assay on SD rats

Male SD rats were anesthetized using diethyl ether. Their dorsal hair was shaved as clean as possible and the skin sterilized using 70% ethanol. A 9-cm by 8-cm square of dorsal skin was further depilated using 8% sodium sulfide. The bare skin was sterilized using 1% povidone iodine solution and cleaned with 0.9% NaCl. Four circular (2.5 cm^2^) second degree burns were produced with YLS-5Q (Biowill Co, LTD. Shanghai, China) on the dorsal skin of each SD rat. The burn device was set at 80°C and applied for 8 seconds. After surgery, each wound was treated for 5 min with either 100 μL of saline, S-FGF (100 ng FGF-1), or 100 μL of T-SM or WT saline aqueous extract for 5 min. The wound was then covered with sterile gauze. After treatment, each rat was housed in an individual cage, 16 × 16 × 9 cm. The burn area was treated once for 5 minutes every other day for 21 days. During the experimental period, all the rats had free access to fresh water and pelleted feed. The wound surface area was determined using the transparent tracing method described by Bohannon and Pfaller [38]. Wound area = (7.5 cm × 7.5 cm × paper weight)/0.1604 g. The area of each wound was recorded on day 3 (n = 15), 7 (n = 15), 14 (n = 12) and 21 (n = 9). Results are expressed as mean ± standard error. Student’s t test was used to evaluate significance differences between groups at p < 0.05.

### Histological staining

Two rats were sacrificed on the day or one day after the burn was administered to determine the severity of the burn. Three rats were euthanized after 7-, 14-, and 21 d of treatment with the different solutions (Saline, S-FGF, T-SM and WT). Tissues including and surrounding the burn wounds were removed and frozen in liquid nitrogen. Wound tissues were cut and processed into 2-μm thick cryocut sections. The effect of different treatments on the wound healing process were determined histologically using a hematoxylin (H) and eosin (E) staining kit (Huaxia Bio Co., Beijing, China). The status of the wound was determined by the presence of inflammatory cells, necrotic cells and tissue, epidermal appendages, granulation tissue, and angiogenesis. Images were taken in the center of each histological section at a 200× magnification.

## Abbreviations

FGF: Fibroblast growth factor; CAM: Chicken embryo chorioallantoic membrane assay; MTT: Thiazolyl blue tetrazolium bromide colorimetric assay; TCM: Traditional Chinese medicine.

## Competing interests

The authors declare that they have no competing interests.

## Authors’ contributions

YT carried out the gene cloning, participated in the plant transformation, experiments and drafted the manuscript. KW designed and analyzed data, and helped to write the paper. NW and GL participated in experiments. DL conceived of the study, and participated in its design and coordination and helped to draft the manuscript. All authors read and approved the final manuscript.

## Supplementary Material

Additional file 1: Table S1 Analysis of recombinant FGF-1 accumulation in the selected transgenic *S. miltiorrhiza* lines. Recombinant FGF-1 accumulation levels in aqueous extract from transgenic lines were analyzed by ELISA. The yield was calculated by the standard FGF-1 curve (Y = 0.0042X + 0.1418). Y is the protein measurement of OD_650_ in 100 μL of 1XPBS buffer. FW: fresh weight of the leaf. The detailed method was described in material and method. Data represents mean of duplicates. Click here for file

Additional file 2: Figure S1 Southern blot. DNA hybrization assay using *fgf-1* as a probe. Genomic DNA (30 μg) from wild-type (WT) and the transgenic (T117) plants was digested with either, *HindIII* or *EcoRI* restriction enzymes. Lane 1, wild type. Lane 2–9, transgenic lines. Lane 1, *fgf-1* PCR product; Lane 2, WT/*HindIII*; Lane 3, WT/*EcoRI*, Lane 4, T117/*HindIII*; Lane 5, T117/*EcoRI*. Click here for file

Additional file 3: Figure S2 Deep-second burn degree determined by H&E analysis. Normal skin had an intact cuticle, hair follicles, sebaceous glands, collagen and a prickle cell layer (A and B). Epidermal necrolysis was observed 24 h after burn treatment (C and D). Micrographs were taken at 200×. Click here for file
