# Supplementary material for: Ectopic expression of human acidic fibroblast growth factor 1 in the medicinal plant, Salvia miltiorrhiza, accelerates the healing of burn wounds
Source: BMC Biotechnol. 2014 Aug 9;14:74. doi: 10.1186/1472-6750-14-74 (PMC4134118; doi:10.1186/1472-6750-14-74)
Supplement: Additional file 1: Table S1 — Analysis of recombinant FGF-1 accumulation in the selected transgenic S. miltiorrhiza lines. Recombinant FGF-1 accumulation levels in aqueous extract from transgenic lines were analyzed by ELISA. The yield was calculated by the standard FGF-1 curve (Y = 0.0042X + 0.1418). Y is the protein measurement of OD650 in 100 μL of 1XPBS buffer. FW: fresh weight of the leaf. The detailed method was described in material and method. Data represents mean of duplicates. [file 1472-6750-14-74-S1.docx]

**Additional file 1: Table S1** Analysis of recombinant FGF-1 accumulation in the selected transgenic *S. miltiorrhiza* lines.

| Transgenic lines | OD_650_ | FGF-1 Yield  (ng/g FW) |
| --- | --- | --- |
| T65 | 0.197 | 131 |
| T97 | 0.203 | 146 |
| T117 | 0.256 | 272 |

Recombinant FGF-1 accumulation levels in aqueous extract from transgenic lines were analyzed by ELISA. The yield was calculated by the standard FGF-1 curve (Y=0.0042X + 0.1418). Y is the protein measurement of OD_650_ in 100 µL of 1XPBS buffer. FW: fresh weight of the leaf. The detailed method was described in material and method. Data represents mean of duplicates.
