# Supplementary figures and images for: Ectopic expression of human acidic fibroblast growth factor 1 in the medicinal plant, Salvia miltiorrhiza, accelerates the healing of burn wounds
Source: BMC Biotechnol. 2014 Aug 9;14:74. doi: 10.1186/1472-6750-14-74 (PMC4134118; doi:10.1186/1472-6750-14-74)

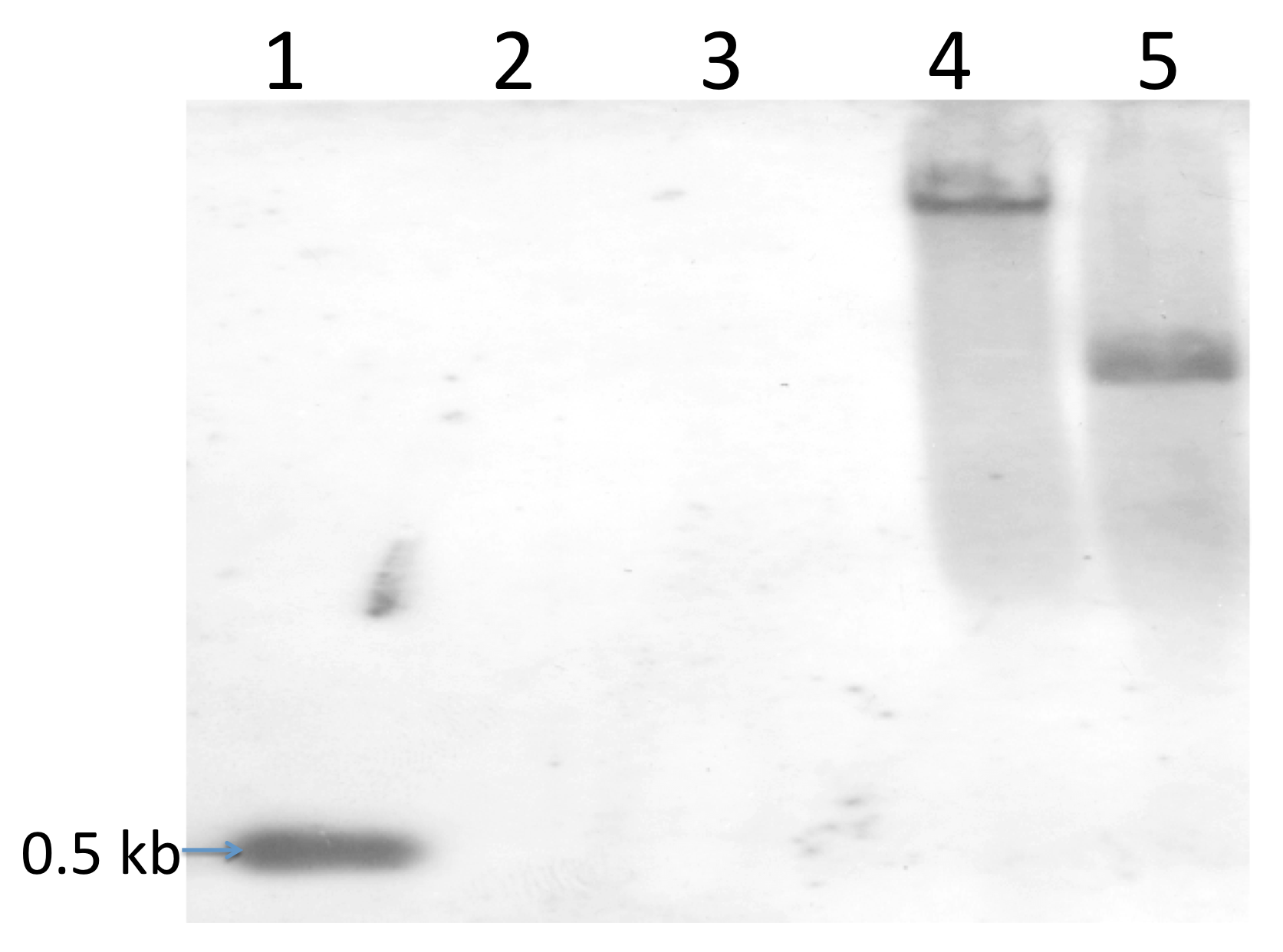

Supplement: Additional file 2: Figure S1 — Southern blot. DNA hybrization assay using fgf-1 as a probe. Genomic DNA (30 μg) from wild-type (WT) and the transgenic (T117) plants was digested with either, HindIII or EcoRI restriction enzymes. Lane 1, wild type. Lane 2–9, transgenic lines. Lane 1, fgf-1 PCR product; Lane 2, WT/HindIII; Lane 3, WT/EcoRI, Lane 4, T117/HindIII; Lane 5, T117/EcoRI. [file 1472-6750-14-74-S2.tiff]

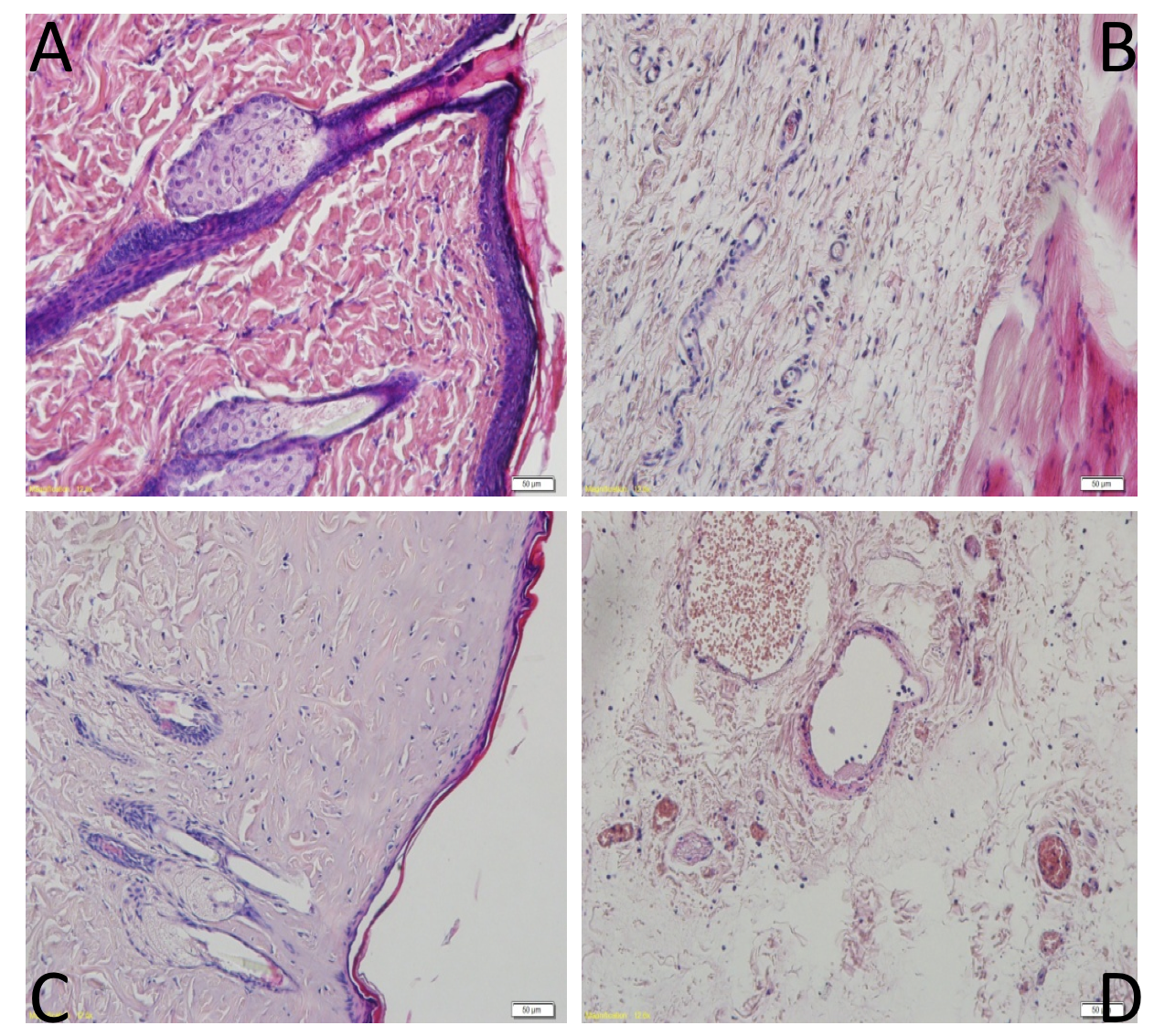

Supplement: Additional file 3: Figure S2 — Deep-second burn degree determined by H&E analysis. Normal skin had an intact cuticle, hair follicles, sebaceous glands, collagen and a prickle cell layer (A and B). Epidermal necrolysis was observed 24 h after burn treatment (C and D). Micrographs were taken at 200×. [file 1472-6750-14-74-S3.tiff]
